# Modulation of V1 Spike Response by Temporal Interval of Spatiotemporal Stimulus Sequence

**DOI:** 10.1371/journal.pone.0047543

**Published:** 2012-10-16

**Authors:** Taekjun Kim, HyungGoo R. Kim, Kayeon Kim, Choongkil Lee

**Affiliations:** 1 Department of Psychology, Seoul National University, Kwanak, Seoul, Korea; 2 Program of Brain Science, Seoul National University, Kwanak, Seoul, Korea; The University of Plymouth, United Kingdom

## Abstract

The spike activity of single neurons of the primary visual cortex (V1) becomes more selective and reliable in response to wide-field natural scenes compared to smaller stimuli confined to the classical receptive field (RF). However, it is largely unknown what aspects of natural scenes increase the selectivity of V1 neurons. One hypothesis is that modulation by surround interaction is highly sensitive to small changes in spatiotemporal aspects of RF surround. Such a fine-tuned modulation would enable single neurons to hold information about spatiotemporal sequences of oriented stimuli, which extends the role of V1 neurons as a simple spatiotemporal filter confined to the RF. In the current study, we examined the hypothesis in the V1 of awake behaving monkeys, by testing whether the spike response of single V1 neurons is modulated by temporal interval of spatiotemporal stimulus sequence encompassing inside and outside the RF. We used two identical Gabor stimuli that were sequentially presented with a variable stimulus onset asynchrony (SOA): the preceding one (S1) outside the RF and the following one (S2) in the RF. This stimulus configuration enabled us to examine the spatiotemporal selectivity of response modulation from a focal surround region. Although S1 alone did not evoke spike responses, visual response to S2 was modulated for SOA in the range of tens of milliseconds. These results suggest that V1 neurons participate in processing spatiotemporal sequences of oriented stimuli extending outside the RF.

## Introduction

**Figure 1:**
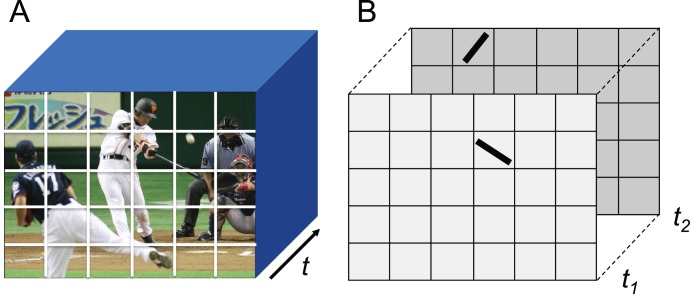
An image volume. A: Spatiotemporal volume of an exemplary visual world. Each rectangle represents a topographically organized unit space corresponding to known receptive field of a single neuron of central visual system such as V1. B: Bars represent oriented line segment of simplified contours of visual events such as a swinging bat at instantaneous moments, *t_1_* and *t_2_*.

The visual world is full of events laid out in space and time. Identifying where and how spatiotemporal relations of event features are encoded in the brain is critical for understanding central visual processing. Imagine that you are watching a video screen in which a baseball player hits a ball (Fig. 1A). Understanding the video can be accomplished by the recognition of spatial features at a given instant and by subsequent detection of changes in static features across time to derive full motion [1]. Physiological evidence bearing on contour integration support reconstruction of object models at a given time frame; for example, the response magnitude of V1 single neurons modulate depending on detection of line segments belonging to a common contour that were simultaneously presented inside and outside RF [2]. Additionally, perceptual organization of image volume can be based on discovering and organizing elementary relations of spatiotemporal sequences before object recognition is completed at a given instant [3]. To apply these ideas to the early visual system, further imagine that a static contour at a given instant is discretized by spatially-confined and oriented filters, such as the classical receptive fields (RFs) of V1 neurons. The video world is now represented as a spatiotemporal volume in which each contour segment exists over space and time with a changing orientation. In this volume, oriented bars at different spatial locations at times *t_1_* and *t_2_* can represent a contour sequence of a common object, for example the bat, discretized by RFs at different times (Fig. 1B). An oriented and discretized feature at *t_1_* can be first integrated with other discretized features at *t_1_* for reconstructing an object contour at *t_1_*, or alternatively, it can be first integrated with another feature at *t_2_* into a spatiotemporal sequence, and then based on resulting sequences, objects and their global motions (as opposed to local motions that are confined within RFs) are simultaneously derived. Note that the combination of locations, orientations and temporal interval of the two oriented stimuli constitutes a unique spatiotemporal sequence. The anatomical sites for processing global motion from spatiotemporal sequence stimuli are not known [4].

Previous studies on the response of V1 single neurons to naturalistic video indicated that the selectivity and reliability of V1 responses increased when a wide-field stimulus simultaneously stimulated zones inside and outside the RF [5], [6]. These results indicate that response selectivity is not fully manifested by RF stimulation, and suggest that V1 neurons are selective for spatiotemporal relations of event features distributed inside and outside the RF. It is not known what aspects of the spatiotemporal relations of event features increase the selectivity of V1 neurons. One requirement for correctly representing the relationship between visual stimuli that are separated in space and time is the encoding of temporal intervals between spatial events. In the current study, we directly examined the effects of varying the temporal interval between sequential stimuli on the spike activity of V1. For this, we confined the priming stimuli to focal zones outside the RF, and presented them asynchronous with the RF stimulus. The response magnitude of single neurons is related to spatial features of stimuli, such as size, spatial location, orientation and contrast. The temporal interval may add another dimension making coding process more complex. As a first attempt, we focused on the activity modulation by the temporal interval between two sequential stimuli of same size, orientation and contrast. The dependence of V1 activity on the temporal interval between sequential visual targets inside and outside the RF would support the hypothesis that V1 neurons are selective for spatiotemporal relations of visual events inside and outside the RF. We varied the stimulus onset asynchrony (SOA) of two identical Gabor stimuli, S1 and S2, presented sequentially at spatially separated positions, while we recorded the spike activity of a neuron whose RF coincided with the second stimulus, S2. The S1, by definition of the RF, does not elicit spiking response of the cell as long as the S1 stimulus lies outside the cell’s RF. However, the response of the cell to S2, which is optimal in orientation, size and location for the cell, is modulated by S1, as previous studies on surround interaction have shown [7], [8], [9], [10], [11], [12]. In this condition, we asked whether the spike activity of V1 neurons in response to S2 is modulated by S1 for SOA in the range of tens of milliseconds corresponding to the physiological range of apparent motion. Previous studies reported that response modulation induced by a surround target enclosing the RF arises with the same latency and monotonically decays with SOA [13]. The response modulation by a RF-enclosing surround stimulus is likely to be made by combined contribution from multiple focal sites within the RF-enclosing zone. Therefore, to investigate whether or not response modulation from focal surround zones also monotonically decays with SOA is a critical step to test the hypothesis that V1 is involved in processing of spatiotemporal sequences of oriented stimuli inside and outside the RF. If the hypothesis is true, response modulation will vary with SOA in non-monotonic ways. Furthermore, if V1 is participating in encoding spatiotemporal configurations of sequential stimuli, response modulation is not only expected to be variable across SOA in non-monotonic ways, but it also to depend on S1 position and orientation, because a unique stimulus sequence is defined by the orientation and position of S1 with respect to S2 as well as the temporal interval between the two stimuli. As a part of sequence stimulus, a focal surround stimulus appeared first and the effects of a variable SOA prior to RF stimulus was measured in the current study.

We found that the activity of single V1 neurons in behaving macaques responded with a magnitude that varied with SOA in non-monotonic ways, to an RF stimulus that followed a focal stimulus outside the RF. These results suggest that such modulation can be used as a clue to resolve temporal intervals between stimuli. In a study dealt with elsewhere [14], we further developed this idea and tested the relationship between response and animal’s behavior in an interval discrimination task (see Discussion).

## Methods

### Ethics Statement

Two male rhesus monkeys (DC and CR, 6–7 years old) participated in the current study. All surgical, experimental, and animal care procedures were approved by the Seoul National University Animal Care and Use Committee, and conformed to the U.S. National Institutes of Health guidelines. Ethical standards incorporated in these procedures include an environmental enrichment program consisting of routine contacts with other animals, expanded cage, regular veterinary care and tests provided by a dedicated personnel, and pharmacological aid ameliorating suffering associated with surgical procedures. These animals were housed in a dedicated colony maintained at a constant temperature and humidity and circulated with HEPA filtered-air. They were fed twice a day with sterile primate diet (Harlan Lab, USA) supplemented with bananas and apples. Aseptic surgical procedures required for neural recording, and anesthetics and analgesics used are described in detail elsewhere [15]. None of these animals were sacrificed for completion of the current study.

### Experimental Procedures

The animals were prepared for chronic extracellular recording and for eye tracking with the scleral search coil technique [16], as described in detail elsewhere [15]. After recovery from the surgery, the animals were trained with their heads restrained to make saccadic eye movements toward a visual target presented on a computer monitor.

Details of experimental procedures were previously described [15]. Briefly, a computer serviced two monitors: one for presenting stimuli and the other for controlling the experimental paradigm. The stimuli were presented on a 24-inch flat CRT monitor (Sony GDM-FW900, 800×600 pixel at a refresh rate of 100 Hz, luminance nonlinearities corrected) by computer programs written in Matlab (The Mathworks Inc.) using the Psychophysics Toolbox [17], [18]. Another computer stored and displayed the data related to eye position, neural activity, experimental status, and the output of a photodiode facing the stimulus monitor. All these signals were digitized at 25 kHz with a resolution of 16-bits (NI-DAQ PCI 6013, National Instruments) with the aid of the DAQ Toolbox (The Mathworks Inc.). This computer communicated with the first at the start and end of each trial in TCP/IP. Timing information was checked off-line against the data stored in the second computer. All timing information described in this report is based on the data stored in the second computer.

Extracellular single-unit activity was recorded from V1 with quartz-insulated platinum-tungsten microelectrodes (Thomas Recording, Germany) advanced through a guide tube. The electrode typically had an impedance of 1–4MΩ at 1 kHz. The guide tube was lowered through the craniotomy until it just contacted the dura. Melted agarose (Agarose LE, SeaMatrix, Korea, 1.5% in saline) was cooled to 37deg and applied around the guide tube to protect the electrode tip and to help recording stability. The electrode penetrated the dura, which had been thinned prior to each recording session. Single neurons were isolated based on peak-to-peak amplitude and duration of spike waveforms during unit recording. A more rigorous classification was performed during off-line analysis based on principal component and cluster analyses of spike waveforms [19] and the presence of refractory period.

For each isolated cell, the RF position and size were first estimated with a Gabor stimulus. The optimal Gabor stimulus was then quantitatively determined while the monkey participated in a simple fixation task. RF size was taken as the diameter of the circular Gabor stimulus producing maximal activity in a spatial summation test [20]. When the neural response did not saturate with increases in stimulus diameter, the largest stimulus diameter among those tested (2 deg) was taken as the diameter of RF. The size of the RF determined this way is usually larger than that estimated with stimuli eliciting minimal responses [7], [21], [22].

**Figure 2:**
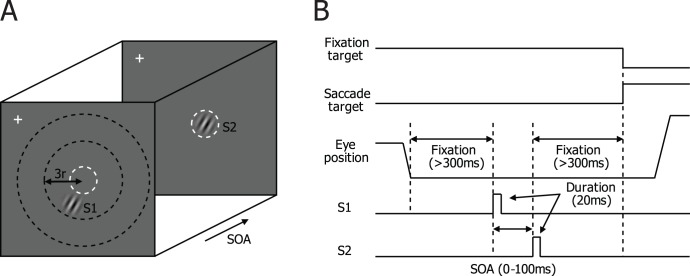
Trial paradigm. (A) A spatial layout of stimulus condition. A white cross indicates central fixation and the dashed white circle (invisible to the animal) represents the classical receptive field (RF). While the eye position was maintained within a window of 1 deg in radius centered about the fixation point, a static Gabor stimulus, S1, was first presented outside RF, and a second static Gabor stimulus, S2, was presented within RF. Both were presented for 20 ms each with a varying stimulus onset asynchrony (SOA), ranging from 0 to 100 ms. The animals’ task was to maintain central fixation and make a saccade following the target for liquid reward. (B) Temporal sequence of a trial.

The main experimental trial started with a beep. While central fixation was maintained within a 2-deg diameter circular criterion window centered on the fixation target, two identical circular Gabor stimuli were sequentially presented, each for 20 ms. The first stimulus (S1) was presented outside the RF and the second (S2) coincided with the RF (Fig. 2). Determination of the boundary between the RF center and surround is not simple [20], [23], [24]. To ensure that S1 did not encroach on the RF, we ensured that S1 did not evoke a spike response. The stimulus onset asynchrony (SOA) varied between 0 and 100 ms in steps of 10 ms. An SOA of 0 ms indicates simultaneous presentation of the two stimuli. To minimize saccade-related activity, the animal had to maintain central fixation for more than 300 ms before S1 onset.

The spatial distance between S1 and S2 was specified in units of RF diameter, center to center. If the radius of the RF is *r*, the area of an S1 at one RF diameter away is one eighth the area of the smallest surround annulus (indicated by the middle concentric circle in Fig. 2A), and that of an S1 at two RF diameters away is one twenty-fourth the surround zone (indicated by the outermost circle). Thus, only a small focal region of the surround is stimulated by S1 in this paradigm. The S1 orientation was the same as the S2 orientation, either collinear or parallel to the S2 orientation, depending on the relative location of S1. Note that each combination of S1 position and SOA constituted a unique S1–S2 sequence, and for sequences, S2 fell on the RF and was the preferred stimulus in terms of position and orientation for a given cell. Only one stimulus condition was tested during central fixation in each trial (Fig. 2B).

In a later phase of the experiments, the positions of stimuli were adjusted to compensate for small movements of gaze direction during fixation in order to stimulate the same retinal location. For this, mean horizontal and vertical eye positions during the 30 ms prior to S1 or S2 onset were calculated and used to determine the physical location of S1 or S2, respectively. This reduced the variability of neural response to visual targets. The variability in the physical location of the stimuli when compensating for gaze movements during fixation was within one deg.

If the monkey maintained central fixation for a variable interval (300–500 ms) after S2 offset, the fixation target went off and the saccade target came on at one of four randomly-chosen positions (left, right, up, and down). This saccade task was used to maintain animal’s concentration. A juice reward was delivered after a correct saccade made within 1 s. The total number of stimulus conditions (thus trials) within a block was determined by the combination of S1 positions and the levels of SOA. In addition, control trials with S1-alone and S2-alone presentations were interleaved among S1–S2 sequence trials. Each stimulus condition was repeated about 20 times in a pseudorandom sequence. Aborted trials with unsuccessful fixation were repeated at the end of each block. We will use brackets to indicate stimulus sequences. For example, [S1a, 60, S2] means S1 was presented at position *a*, followed by S2 with SOA of 60 ms.

The background monitor luminance was either dark (0.00 cd/m^2^) or gray (1.79 cd/m^2^). The mean luminance of the stimulus was higher than that of background to reduce visual reafference signals from the monitor edge associated with saccadic eye movements.

### Data Processing

The data from invalid experiments and trials were excluded from further analyses. Data for which the cell showed a spiking activity to S1 stimulus were excluded. These cases were due to a partial overlap in the spatial extent between S1 and the RF of the cell under study, despite the estimation of RF size with the spatial summation test. In the remaining experiments at 126 sites recorded from two monkeys, S1 alone evoked virtually no spikes; mean firing rate during the interval between 50 and 150 ms following S1 onset was, on average, 3.53% that of S2 alone in these sites. In the data collected during the earlier phase of experiments in which gaze-dependent stimulus presentation was not applied, the response magnitude was relatively more variable due to the variability of eye position during fixation. Thus, the trials in which the eye deviated more than 0.5deg from the fixation target, or the peak instantaneous eye velocity exceeded 40 deg/s during target presentation were excluded. Also, within each stimulus condition, the trials in which the mean firing rate during the interval between 50 and 150 ms following S2 onset exceeded two standard deviations from the mean were excluded. Trials in which the output of the photometer facing the stimulus monitor was in conflict with the intended stimulus duration or SOA were also discarded; these comprised less than 0.3% of the collected trials.

### Analysis Time Window

We frequently observed that the RF stimulus (S2) evoked strong transient activity followed by weak sustained neural activity. Since the latency and duration of neural activity in response to a brief stimulus were variable across cells, we used a variable analysis window. The onset and offset of the analysis time window were defined from the S2 alone condition as the first and the last time at which spike density crossed the half maximum response level, 0.5×(*r_peak_ − r_baseline_*), where *r_peak_* is peak firing rate and *r_baseline_* is baseline activity obtained from the mean firing rate during the interval from −200 ms to −100 ms relative to stimulus onset, averaged over all trials. The mean start time of the analysis window was 68.44 ms after target onset, and its mean duration was 63.14 ms. This window was used to compute both response index and selectivity index described below.

### Numerical Index for Response Magnitude

In order to quantify the magnitude of response modulation by S1, a *response index* was defined for each SOA condition of each S1 position as (*r_1–2/_r_2_* ) ×100, where *r_1–2_* is the mean spike density for the S1–S2 sequence stimulus of that SOA, and *r_2_* is the mean spike density for S2 alone. Thus, the magnitude of response to the S1–S2 sequence stimulus is expressed as a percentage response; a response index of 100% indicates no effect of S1, and a response index larger or smaller than 100% indicates facilitation or suppression by S1 of the response to S2 alone, respectively, at a given SOA.

### Analysis of Temporal Selectivity

The magnitude of response modulation across the temporal intervals between S1 and S2 was quantified with the *selectivity index* [6], [25],



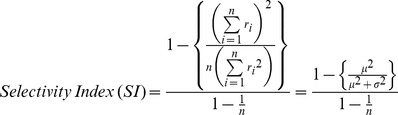
,where *n* is the number of SOA conditions, *r_i_* is the mean response in the *i^th^* SOA condition, and *ì* and *ó* represent the mean and standard deviation of response, respectively. If a cell were nonselective, the activity would be constant across SOA making the numerator and denominator of the terms in braces equal, and the selectivity index would be *0*. In contrast, if a cell responds only in one SOA condition and is silent in all other SOA conditions, the ratio in braces becomes *1/n*, and the selectivity index becomes *1*. A bootstrap method was used to test the significance of the selectivity index for each cell. For this, the probability distribution of the selectivity index was derived for each cell by randomly shuffling trials from all SOA conditions. The null hypothesis was that all SOA conditions have the same mean firing rate, and thus the selectivity index is zero. For each neuron, the probability distribution of the selectivity index under the null hypothesis was made from 1000 simulated experiments.

### Time Course of Facilitative and Suppressive Modulation

As will be described in the following text, modulation of neural response to S2 by the preceding S1 was time-varying. Occasionally, the modulation was initially suppressive, but later changed to facilitative. In order to capture this time-varying modulation, we computed 30 ms-moving averages in steps of 5 ms. At each epoch, we performed nonparametric test (Mann-Whitney U-test) between the magnitudes of activity in S2 alone and S1–S2 conditions. This procedure was repeated for each SOA condition and compiled together across trials and SOA to visualize the pattern of significant modulation as function of both SOA and poststimulus time.

## Results

### Data Summary

We recorded spike activities at a total of 126 recording sites in three hemispheres of two monkeys while they participated in a simple fixation task in which two identical Gabor stimuli were sequentially presented, each for 20 ms (Fig. 2). The current report is based on 49 single cells and 77 multiunit activities recorded from these sites. For each site, single or multiple S1 positions were tested, and analyses were carried out for 276 stimulus conditions in total (Table 1). We recorded from the operculum of V1, typically taking data on the first encountered cell with clear visual driving and well-defined waveforms. Hence most neuronal data were probably collected from layers 2 and 3. The median eccentricities of RF centers were 3.32 deg for the left hemisphere of monkey DC (31 sites), 3.57 deg for the right hemisphere of monkey DC (28 sites), and 6.53 deg for the left hemisphere of monkey CR (67 sites). The median RF diameters were 1.6, 1.6, and 1.8 deg for these three hemispheres, respectively. Except when stated otherwise, results from the two monkeys were similar and were combined in the following analyses.

**Table 1:** Summary of stimulus conditions.

S1–S2 Distance in RF units	Ipsilateral S1∶227 (8)		Contralateral S1∶49 (8)		Total: 276 (8)	
	Collinear	Parallel	Collinear	Parallel	Collinear	Parallel
1	122 (8)	21	27 (8)	5	149 (16)	26
2	48	21	10	5	58	26
3	15	0	2	0	17	0
Total	185 (8)	42	39 (8)	10	224 (16)	52

Ipsilateral S1: S1 was presented in the hemifield ipsilateral to RF; Contralateral S1: S1 was presented in the hemifield contralateral to RF. The numbers in parentheses refer to stimulus conditions in which the response to S2 alone was not tested.

### Neural Response to Sequence Stimuli

**Figure 3:**
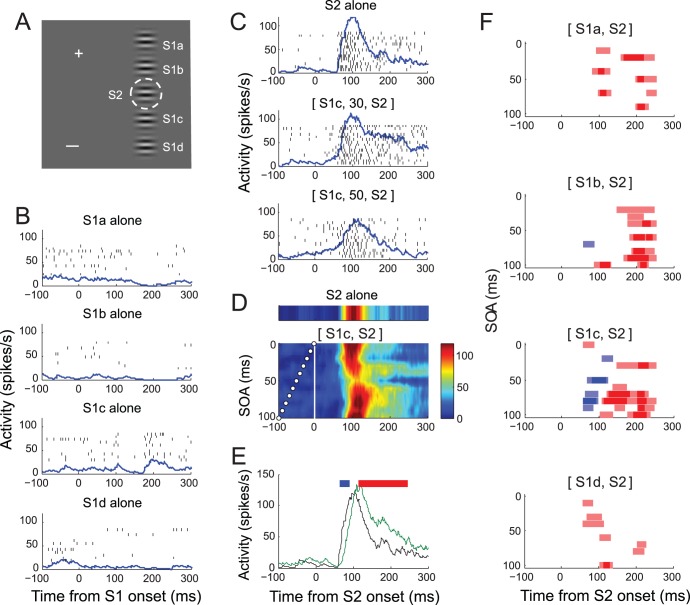
Response of a representative cell. (A) Spatial relation between stimuli in screen coordinates (calibration bar = 1 deg). White cross represents fixation target, and the dashed circle (invisible to the animal) encloses the RF of the recorded neuron determined with a spatial summation test. Gabor stimulus at RF (S2) is at preferrred orientation. S1 was presented at one of four locations, a–d, along the axis orthogonal to that of RF orientation, with a spacing of one RF diameter. All S1 orientations were parallel to S2. There were 44 unique stimulus sequences (4 S1 positions×11 SOAs), plus five single stimulus conditions at each S1 and S2 locations. These 49 stimulus conditions were randomly repeated. (B) Raster and density plots of response to S1 at positions a–d aligned at its onset. Spike density function was derived by convolving spike sequence with an asymmetric kernel function [66]. Y-axis indicates spike density in spikes/s. Note that no S1 alone at positions a-d evoked spike response. (C) Raster and density plots for S2 alone and S1c-S2 sequence stimuli with SOAs of 30 and 50 ms chosen to illustrate response modulation. Trials are aligned at S2 onset. It can be seen that the magnitude of initial and sustained response varied with SOA. (D) An example SOA-time plot compiled from spike density for S1c-S2 sequence stimuli, the first stimulus at positions c and the second stimulus at RF. Y-axis is SOA, determined in 10-ms step. The times of S1 onset for each SOA condition are indicated as small white circles. Data are linearly interpolated across SOA. The S2-alone condition is given above for comparison. Note that the cell’s response varied with SOA. (E) Determination of significant modulation. Spike density curves for S2 alone (black) and S1c-S2 sequence with SOA of 80 ms (green), along with horizotal marks (top) of temporal epochs associated with statistically significant decrease (blue) and increase (red) from S2 alone condition. (F) Time course of significant modulation of spike response by sequence stimuli as shown in E. Spike density following S1–S2 sequence was compared with spike density following S2 for each of temporal epochs of 30 ms with a shift of 5 ms. The temporal epochs with a statistically-significant decrease in spike density as determined with Mann-Whitney U-test are shown in blue bars, and significant increase in red bars, centering on corresponding analysis windows, revealing the magnitude and time course of suppressive and facilitative effects of S1 that depend on S1 position and SOA. The dark symbols represent significant modulation at p<0.01, and the light ones are p<0.05.

Fig. 3 illustrates the activity of a representative V1 cell recorded during the task. For this cell, S1 was presented at one of four positions, *a* through *d*, along the axis perpendicular to the orientation of S2 (Fig. 3A). S1 alone evoked no spike responses at any of these four positions, verifying that these stimuli were presented outside the RF (Fig. 3B). In contrast, S2 alone evoked a vigorous spike response that started at around 50 ms, peaked at around 100 ms after S2 onset, and decayed thereafter (upper panel of Fig. 3C). When S1 and S2 were sequentially presented, S1 did modulate the cell’s response to S2 in a manner that varied with SOA. For example, when S1 was presented 30 ms prior to S2, the response became more sustained (middle panel of Fig. 3C), whereas with an SOA of 50 ms, the peak response was considerably reduced (lower panel of Fig. 3C).

We refer to the modulation of spike response as a function of SOA as *SOA-dependency,* in the sense that response modulation was not constant across SOA. In order to visually examine SOA-dependency, we first sorted valid trials according to SOA, calculated a spike density function for each SOA condition, and then derived the SOA-time plot by compiling spike density functions across SOA conditions with a color code (Fig. 3D). Notably for this cell, the response was strongly suppressed by S1c at an SOA of 50 ms (Fig. 3D). At some SOAs, response modulation by S1 was both facilitative and suppressive, depending on the temporal analysis window; an example illustrated in Fig. 3E shows that with an SOA of 80 ms, S1c suppressed the initial response, but subsequently facilitated the response compared to the S2 alone condition. Alternatively, the effect can be described as a delayed response. The precise pattern of response modulation by S1, thus greatly varied depending on SOA. Non-parametric tests were performed on spike density functions for defining epochs of statistically significant modulation, as shown with blue (suppression) and red (facilitation) horizontal bars in Fig. 3E.

Similar analyses were repeated for S1 at other locations and SOA-time plots were derived for each S1 location (not shown). When the cell’s response during presentation of the S1–S2 sequence was compared with the response to S2 alone, the effect of adding S1 at positions *a* or *d* was facilitative at selective SOAs (red color in Fig. 3F), whereas S1 at positions b or c was both suppressive (blue) and facilitative (red). The pattern of the time course of SOA-dependent significant modulation was complex, and apparently depended on the combination of S1 position and SOA; for the same S1, modulation varied with SOA, and for the same SOA, modulation varied with S1 position.

**Figure 4:**
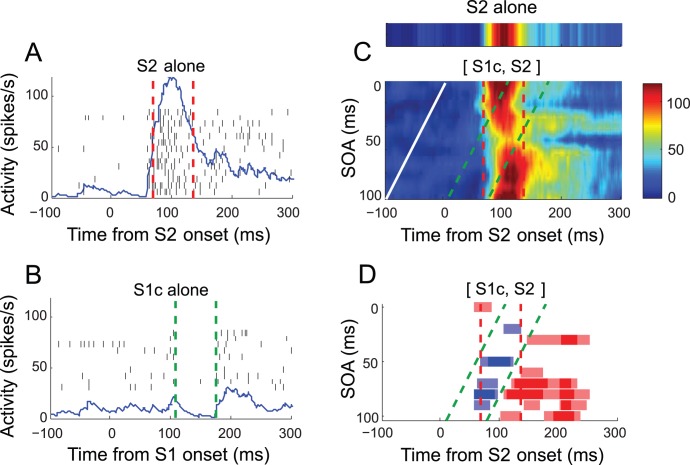
Modulation window. The cell of Fig. 3 is reproduced. (A) Response to S2 alone with the duration of 63 ms for inital transient response indicated with red dotted lines. (B) The duration of suppression by S1 at the position c is indicated with two green dotted lines. (C, D) Modulation window formed by the two durations in A and B in corresponding colors. Time of S1 onset is shown in a white line, interpolated across SOA. The S2-alone condition is given above for comparison in C. Note that the range of significant suppression and facilitation agrees well with modulation window. Also note that modulation is variable depending on SOA within modulation window.

The fact that SOAs were not equally effective in modulating spike response was likely related to the neural latency of S1, i.e., the time it took from presentation of S1 to the start of modulation manifested at the neuron under study. Since S1 alone did not evoke a response, and the suppressive or facilitative modulation was only manifested in the neural response to S2, the modulation occurred only within a time window determined by both the duration of the spike response to S2 and the duration of the S1 effect. Typically, it was difficult to estimate the time course of modulatory effects of S1, but in rare cases, the response to S1 alone caused a suppression of spike activity. This point is illustrated in Fig. 4, reproducing the response of the cell of Fig. 3. The S1 at the position *c* did not excite the cell, but suppressed the spontaneous activity later (Fig. 4B), allowing estimation of suppression duration. Suppression and facilitation in response to the S1–S2 sequence can be partly explained by the sum of the time course of excitatory response to S2 alone (Fig. 4A) and the time course of response to S1 alone (Fig. 4B). The combined time course constitutes a window of response modulation (Fig. 4C). The temporal window for suppressive and facilitative modulation corresponded fairly well to the combined time course (Fig. 4C, D). For example, the decrease in spontaneous activity (green dotted lines) and ensuing increase in Fig. 4B are reflected in the temporal range of significant suppression and facilitation in Fig. 4D. We emphasize, however, that within this window, modulation was not constant, but varied depending on SOA. In other words, response to the S1–S2 sequence was not completely explained by a (weighted) sum of SOA-adjusted individual responses to S1 and S2 alone. For example, in Fig. 4D, within the windows of suppressive and facilitative modulation defined by the combined time courses, the suppression was significant at an SOA of 50 ms, but not for SOA of 40 or 60 ms.

### Neural Response to Collinear Sequence Stimuli

**Figure 5:**
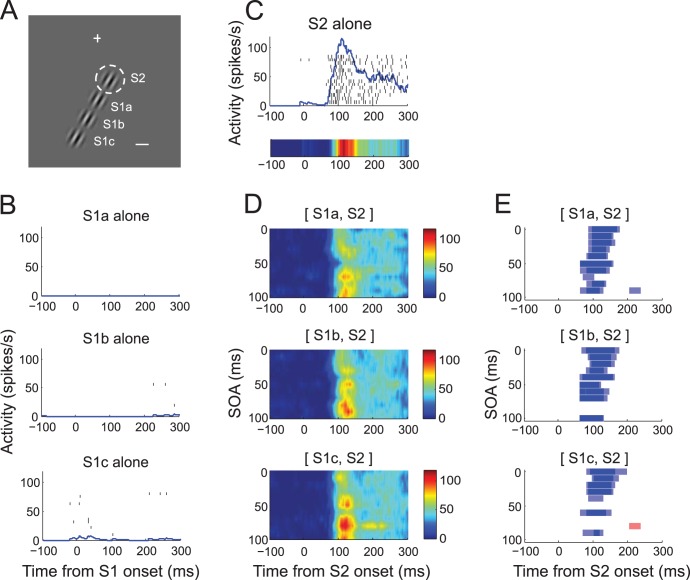
Response of another representative cell. (A) For this cell, S1 was presented at one of three locations, a–c, as shown along the axis collinear to that of RF orientation, with a spacing of one RF diameter. Some S1s encroached on the hemifield contralateral to RF. S1 orientation was collinear to S2. There were 33 unique stimulus sequences (3 S1 positions×11 SOAs), plus four single stimulus conditions at each S1 and S2 locations. These 37 stimulus conditions were randomly repeated within a block. (B) Spike activity with stimulation of S1 alone at locations, a–c. The cell remained silent with S1 at all tested locations. (C) Spike activity with S2 stimulus alone in raster and density (upper) and color (lower) plots. (D) SOA-time plots in the same format as Fig. 3D, for S1 at locations, a–c, from top to bottom. Color map of activity is shown to the right. Note a periodic SOA-dependency of activity modulation (E) Time course of significant modulation of spike response by sequence stimuli in the same format as Fig. 3F for S1 at locations, a–c, from top to bottom. Note that the activity modulation by the S1 at all locations was suppressive at virtually all SOAs. All the stimulus conditions of Fig. 5 were randomized within the same block during data collection.

Fig. 5 illustrates the activity of another representative V1 cell recorded during the task. For this cell, S1 was presented at one of three different locations, *a* through *c*, one to three diameters of RF away from the cell’s RF center along the axis collinear to the cell’s preferred orientation (Fig. 5A). The cell was recorded from the left V1 and its RF lay in the right visual space (dashed circle in Fig. 5A). With the stimulus configuration of Fig. 5A, some S1s were thus presented in the hemifield contralateral to RF, but perhaps partially overlapping with the strip of ipsilateral representation. The S1 alone at none of these positions evoked spike responses, confirming that these stimuli were presented outside the RF (Fig. 5B). The spike activity in response to presentation of S2 alone consisted of a transient increase and gradual decay (Fig. 5C). Although S1 alone did not evoke spike responses, presentation of S1 at any of these positions at the time of S2 presentation modulated the spike response at selective SOAs (Fig. 5D, E). The response modulation by S1 was mostly suppressive for the initial transient response during a poststimulus period of 50–150 ms and varied with the combination of SOA and S1 position. For example, S1 at the position *a* suppressed most strongly at the SOA of 50 ms, but at the same SOA, S1 at *b* or *c* did not suppress as much. Note that this SOA-dependency is a property of V1 cells encompassing both spatial regions inside and outside RF, thus separate from motion tuning or directional selectivity confined within RF.

**Figure 6:**
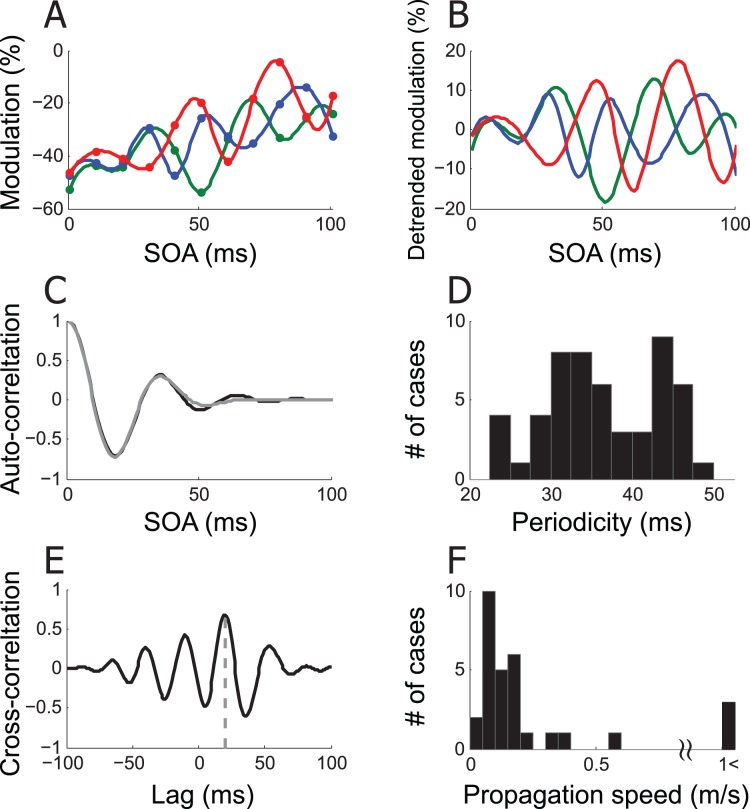
Periodic SOA-dependency of surround modulation. (A) Modulation of spike response in percentage as a function of SOA for three S1 locations of Fig. 5; green: a, blue: b, red: c. (B) Periodic component. The best fit linear trend was removed from the spline fit of modulation percentage in A for each S1 position to remove the monotonic component (detrend.m provided by the MATLAB). (C) The auto-correlogram (black) of the detrended modulation of S1a (green curve of B) was fit with a cosine-Gaussian function, 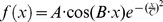 (gray). (D) Hstogram of periodicity. Each case is the first non-zero peak of a cosine-Gaussian function, taken from 53 out of 276 stimulus conditions, for which the cosine-Gaussian fit explained more than 90% variance of auto-correlation curve. For three examples of B, R-squares are 0.99 (S1a), 0.94 (S1b), 0.99 (S1c). The mean periodicity of 53 stimulus conditions is 36.62 ms. (E) Cross-correlation between detrended green and blue curves of B. The time lag at the maximum cross-correlation is 20 ms. This lag reflects the distance between S1a (green) and S1b (blue). Given that cortical distance between S1a and S1b was 3.07 mm, the propagation speed in this example is estimated to be 0.15 m/s. (F) Histogram of propagation speed. Shown is propagation speed for each of 30 cases in which at least one S1 position passed the periodicity criteria of C. The mean distribution is 0.14 m/s without 3 outliers. The median is 0.11 m/s.

The response to S2 was often modulated at periodic SOAs. Fig. 6A illustrates an example of periodic SOA-dependency of response modulation, taken from the cell of Fig. 5. The suppressive modulation in Fig. 6A appears to be consisted of two components; a monotonic and a periodic SOA-dependencies. A monotonic component depends on the spatial proximity of S1 to S2 and on SOA between them. The suppressive modulation for the cell was larger with a closer S1 and at shorter SOAs; compare overall vertical positions of green, blue, and red traces, and compare modulation magnitude between shorter and longer SOAs. In addition to this monotonic component, response modulation was repetitive at multiple SOAs, and appears to be periodic. To quantify the periodic component, the linear trend was first removed (Fig. 6B), and the auto-correlation of the detrended function was fitted with a cosine-Gaussian function (Fig. 6C). The periodicity was taken from the fitted cosine-Gaussian function that explained more than 90% of variance of the auto-correlation function. The mean periodicity of 53 out of 276 stimulus conditions for 45 recording sites (14 single-units and 31 multiple-units) was 36.62 ms (Fig. 6D). This indicates that for these conditions the influence from focal surround regions arrives in a repetitive wave of gamma frequency. This may be related to the fact that stimulus onset generates a neural response in a gamma rhythm [26], [27] based on a network of inhibitory interneurons [28], [29] that propagates and results in modulation of spike response in a gamma rhythm [30].

Another interesting feature seen in Fig. 6A is that the effect of S1 location becomes apparent at long rather than short SOAs. For short SOAs (<20 ms), the magnitude of response modulation was relatively comparable across three S1 conditions. However, for larger SOAs (>20 ms), the magnitude of response modulation varied depending on S1 position resulting in an apparent phase shift (Fig. 6B). This position-phase relation provides an opportunity to estimate the propagation speed of S1 influence. For this, we converted the screen coordinates of S1 positions to anatomical coordinates within the cortical map [31]. The distance between cortical representations of S1a and S1b was 3.07 mm. As an estimate of the temporal delay between surround influences from S1a and S1b, we obtained the time lag of 20 ms that was associated with the maximal cross-correlation between detrended spline approximations for S1a and S1b (Fig. 6E). Based on these measures, the propagation speed of periodic component is estimated to be 0.15 m/s. Similar calculations for S1b and S1c gave 2.56 mm of cortical distance and 21 ms of maximal correlation, resulting in 0.12 m/s. Fig. 6F illustrates the histogram of estimated speed for 30 stimulus conditions in which at least one S1 passed the periodicity criteria, with the mean propagation speed of 0.14 m/s excluding 3 outliers, ranging between 0.04 m/s and 5.37 m/s. These estimates of propagation speed agree well with the previous estimate of the speed of horizontal connection which ranges between 0.05 and 0.5 m/s [32], [33], suggesting that the periodic component is mediated by horizontal connections.

In contrast to the periodic component in Fig. 6A, the response modulation at short SOAs (<20 ms) appear to be similar across three S1 positions. Bair et al.(2003) reported that the onset latency of suppression for distant surround stimulus was rarely delayed than that for nearby surround stimulus, and suggested that feedback from extrastriate cortex cells with larger RFs can account for the suppression. Thus, the modulation at short SOAs may reflect fast feedback connections from extrastriate cortex, whereas the response modulation at larger SOAs may reflect horizontal connections as stated above. The response modulation at short SOAs that is independent of S1 position may reflect large RFs of the cells that provide the feedback signal, and thus a less discrimination of S1 position. In contrast, the same S1 positional variation may cause a bigger change in horizontal connection signal originating from V1 with smaller RFs.

Regarding the above analyses on periodicity, we would like to note the limitation of our method. Since we measured response modulation at the SOA step of 10 ms, the periodicity analysis is inherently limited by this precision, making very fast propagation speed evades our estimates.

### Parallel Versus Collinear Orientation

**Figure 7:**
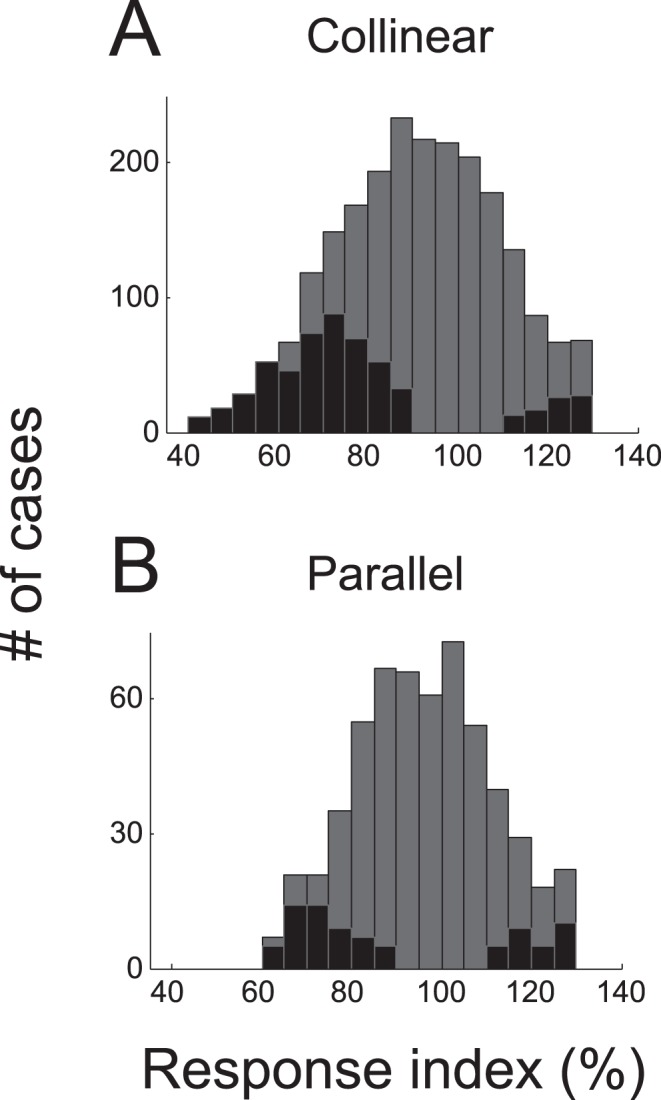
Frequency histograms of response index for each SOA condition from 208 collinear (A), 52 parallel (B). The mean indices were 91.23±18.40 and 96.28±15.24, respectively. These means are significantly less than 100% (Wilcoxon signed-rank test, all p<10^−7^). The proportions of significant suppression and facilitation (black bars) were 21.24 and 3.72% (A), and 9.44 and 5.07% (B), respectively. Note that suppression was more frequent than facilitation, for both collinear and parallel configurations, but this difference was larger for collinear condition.

The effect of S1 was quantified with a *response index*. For each S1, 11 SOA (0 to 100 by 10 ms) conditions were tested, and for each SOA condition, the magnitude of neural response to the S1–S2 sequence stimuli relative to the response to S2 alone was taken as the response index. Fig. 7 illustrates the overall distribution of response index for parallel (as in Fig. 3) and collinear (as in Fig. 5) S1 conditions separately. Overall, the mean response index was 91.23% for collinear and 96.28% for parallel configurations. Both of these measures are significantly less than 100% (Wilcoxon signed-rank test, p<10^−7^) indicating suppression in both configurations. The difference in response index between the two configurations was statistically significant (Mann-Whitney U-test, p<10^−8^), indicating that collinear S1 suppressed activity on average more than parallel S1. In addition, significant modulation differed with stimulus configuration; the ratio of significant suppression to facilitation was 5.71 (21.24% for suppression vs. 3.72% for facilitation) for collinear, and 1.86 (9.44% for suppression vs. 5.07% for facilitation) for parallel configurations, indicating that suppressive modulation was dominant with collinear S1. These results are consistent with previous studies that reported a strong suppression by collinear stimulus at the RF ends [10].

**Figure 8:**
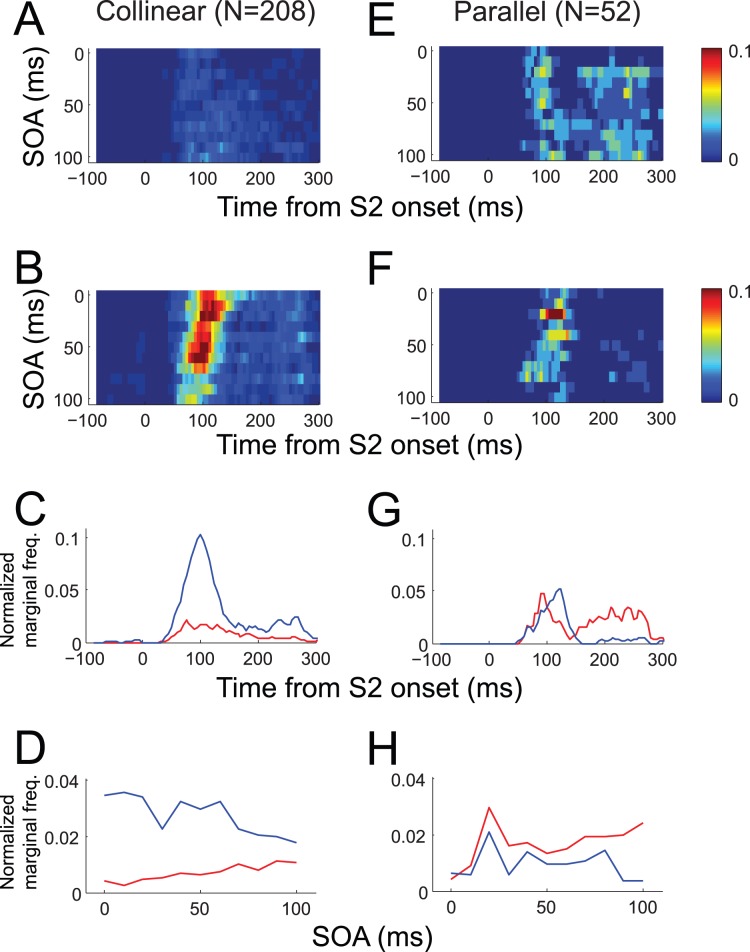
Pattern of modulation for collinear (A–D) and parallel (E–H) S1 conditions. (A) Combined time course of SOA-dependent significant facilitation (p<0.05) from 2288 SOA conditions of 208 collinear S1 stimuli. Normalized frequency of significant epoch is color-coded according to the color map shown on the right. Out of 2288, 208 (9.09%) SOA conditions included more than one temporal epoch with significant facilitation. (B) Time course of significant suppression combined from the same collinear S1 configurations. In 611 of 2288 (26.70%) SOA conditions, more than one temporal epoch showed significant suppression. (C) Normalized marginal frequency of significant facilitation from A (red) and suppression from B (blue) against peristimulus time. (D) Normalized marginal frequency of significant facilitation (A, red) and suppression (B, blue) during the poststimulus time period from 0 to 300 ms against SOA. Normallized marginal frequency was derived from marginal sum divided by the number of data points. (E–H) Similar plots as A–D combined from 572 SOA conditions of 52 parallel configurations. Out of 572 SOA conditions, 96 (16.78%) and 71 (12.41%) SOA groups showed significant facilitation and suppression, respectively. Thus, suppression was relatively common with collinear S1, and the relative ratio of facilitative modulation was higher with the parallel configuration. This was true even after the distance between S1 and S2 was taken into account (by subdividing S1 configuration conditions into two distance groups, one or two RF diameter away from RF center). Note that suppressive modulation was concentrated at around 100 ms after S2 onset time (C, G), whereas facilitative modulation was relatively more dispersed and dominant after around 200 ms after S2 onset, especially in the parallel configuration. Also note that collinear S1 tended to suppress at short SOA and facilitate at long SOA (D), whereas this dissociation was relatively weak with parallel S1 (H).

The sign of surround interaction is known to vary with stimulus contrast [10], [34]. Thus, the precise ratio of facilitative to suppressive interaction may vary. We focus here on the temporal interval rather than the sign of surround interaction, whether or not the incidence of significant suppression or facilitation is constant across SOA. For this, we combined the plots of the time course of SOA-dependent significant modulation, such as Fig. 3F, for suppression and facilitation separately for 260 experimental conditions in which the response to both S1–S2 sequence and S2 alone were tested (Fig. 8). Here again, it can be seen that for collinear S1 conditions, the incidence of significant suppression (Fig. 8B) was much more frequent than that of significant facilitation (Fig. 8A). This difference is not apparent for parallel S1 conditions (compare Fig. 8E and F). The time course of modulation was also different; for collinear S1 conditions, suppressive modulation was concentrated at around 100 ms after S2 onset (Fig. 8C), which corresponded to the time of peak response to S2 alone, whereas facilitative modulation was relatively more dispersed (Fig. 8E). For parallel S1 conditions, facilitation was dominant after about 200 ms following S2 onset.

Suppression and facilitation were not constant across SOA; for collinear S1 conditions, with increases in SOA the incidence of significant suppression tended to decrease (blue trace of Fig. 8D), whereas significant facilitation tended to increase (red trace of Fig. 8D). For parallel S1 conditions, this trend was relatively weak (Fig. 8H). We would like to note that our data were sampled from a limited range of RF location relative to the whole visual field, and from a limited range of S1 positions with respect to RF. Nevertheless, it was clear that tested SOAs were not equally effective in modulating spike response, and that the pattern of SOA-dependency was different between suppressive and facilitative modulation (Fig. 8D).

There was no apparent difference in the tendency of occurrence of periodic SOA-dependency between collinear and parallel S1 conditions; a fitted cosine-Gaussian function explained more than 90% of variance of the auto-correlation function in 44 of 224 collinear S1 conditions (19.64%) and 9 of 52 parallel S1 conditions (17.31%).

### Effects of Spatial Distance between S1 and S2

**Figure 9:**
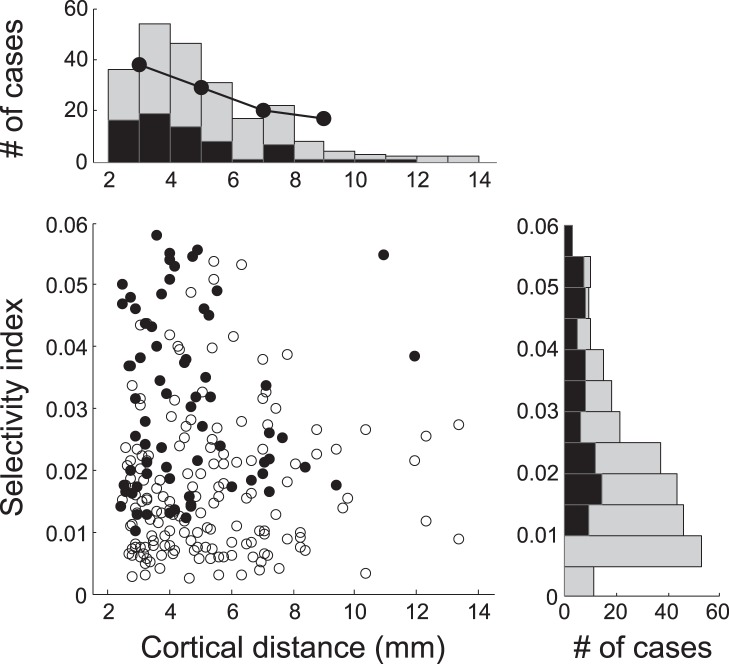
Scatter plot of selectivity index and cortical distance. Each dot represents selectivity index (SI) for each stimulus condition and anatomical distance between the centers of S1 and S2 for that condition. Mariginal histograms are also shown. The cortical distance was estimated from the cortical magnification factor [31]. Data are combined single and multiple unit data obtained from 227 stimulus conditions (i.e., S1 positions) for 105 recording sites in 2 monkeys. Black dots and bars indicate significant *SIs* (69 of 227 cases, 30.40%, p<0.05), as evaluated with a bootstrapping method. The proportion was also consistent for single units alone (23 of 92 stimulus conditions, 25%) and multiple unit activitiy (46 of 135 stimulus conditions, 34.07%).The proportion of significant *SI* decreased with the cortical distance between S1 and S2.

The degree of dependence of response modulation on SOA for each S1 was quantified with *SI*, a numerical index of selectivity of facilitation or suppression for SOA. Overall, *SI* ranged from 0.002 to 0.058 (Fig. 9, right marginal histogram). To give an idea of the meaning of this index, if neural response was reduced by 30% by the S1–S2 sequence stimuli in one of 11 SOA conditions, *SI* would be 0.009, and if it was reduced by 30% equally in 3 of 11 SOA conditions, *SI* would be 0.023. Note that some cells showed near zero *SI*, indicating a lack of interaction with the tested S1. This is not unexpected because we used a focal surround stimulus and previous studies showed that surround interactions originate from specific regions of the surround [11], [35], and because surround interactions are evident only for a subset of V1 cells [36]. *SI* was computed based on the mean firing rate during an analysis time window that was chosen to include strong transient activity centered at about 100 ms after S2 onset, and its significance was judged by a bootstrapping method (see Materials and Methods). When *SI* was computed over moving temporal epochs of 100 ms with a step of 50 ms from −150 ms to 450 ms with respect to S2 onset, the frequency of significant *SI* remained low before S2 onset, began to grow, reached maximal at about 100 ms from S2 onset, and then decreased to baseline level. Thus, the time course of the frequency of significant *SI* resembled the time course of spiking activity evoked by S2.

In order to understand the effects of spatial distance between S1 and S2, we analyzed the proportion of significant selectivity index (SI) as a function of distance in cortical dimension. The anatomical distance between cortical sites representing centers of S1 and S2 that were confined to the same visual hemifield (227 conditions of 105 recording sites including both collinear and parallel configurations) was estimated from the cortical magnification factor [31]. Fig. 9 shows the distributions of the estimated anatomical distance (top marginal histogram). Most of our data were obtained with a S1 whose center position was one RF diameter away from the RF center, which corresponded to a distance of 3–5 mm in the cortex. A bootstrap statistical test revealed that *SIs* for 69 of the 227 conditions (30.40%) were significant (p<0.05, black dots of the scatter plot and bars of histograms in Fig. 9). The proportion of significant *SI* was high at short distances and decreased as the distance between S1 and S2 increased (black dots of top marginal histogram of Fig. 9). Significant *SIs* were found up to a distance of at least 10 mm. Note that the selectivity index underestimates the S1 effects when modulation was delayed, because the index was derived from an analysis window centered on the initial transient response. In 49 S1 conditions, the centers of S1 and S2 were in the opposite hemifield, and the *SIs* from 11 (22.45%) conditions of these were statistically significant. Although these 49 conditions were not included in Fig. 9, surround interaction for these conditions also decreased with distance between the centers of S1 and S2.

As shown in Fig. 9, the incidence of significant *SI* decreased as the distance between S1 and S2 increased. It appears that the pattern of distance-dependency differed for collinear and parallel configurations; significant selectivity index occurred over a relatively larger spatial distance for collinear than for parallel configurations. The proportion of significant selectivity index decreased from 34% (41 of 122 collinear S1s) to 25% (12 of 48 collinear S1s) as the distance between collinear S1 and S2 increased from one to two RF diameters, whereas it decreased from 38% (8 of 21 parallel S1s) to 14% (3 of 21 parallel S1s) with the same increase in distance for parallel S1. These results suggest that the shape of S1 zone for SOA-dependency is not circularly symmetrical, but elongated along the axis collinear to preferred orientation.

### Effects of S1 on Neural Latency

So far, the analyses have focused on the effects of S1 in firing rate. If suppresive inputs temporally overlaps with excitatory inputs, time to spike threshold, i.e., neural latency, as well as firing rate may also be modulated [37]. We defined neural latency as the first time point at which spike density function exceeded 2 standard deviations from baseline level. Firing rate was derived from the mean spike density during the period of 100 ms from neural latency, not from a poststimulus time period, to avoid its ‘false’ estimation [15]. Because the stimulus condition such as S1 or RF location varied across cells of our sample, simply relating firing rate and neural latency of each SOA condition might be of less meaning. Thus, we ordered SOA groups according to mean firing rate or neural latency, and then we examined how the ranks in the two categories were related to each other. The correlation coefficient between ranks was −0.26 (p<10^−4^), indicating that neural latency and firing rate were negatively correlated. When we calculated the mean neural latency for each SOA groups sorted according to mean firing rate, the firing rate overall varied by approximately 30% and neural latency varied by about approximately 4 ms depending on SOA. Thus, S1 changed neural latency as well as firing rate; with a stronger suppression, the neural latency became longer and firing rate became lower.

We also attempted to quantify the degree of SOA-dependency of neural latency for each S1 by using the *SI* equation that was identical with the one described above (for this, *r_i_* is the mean neural latency in the *i^th^* SOA condition). Its statistical significance was evaluated with the bootstrapping procedure introduced above. A bootstrap statistical test revealed that *SIs* of neural latency for 34 of the 227 conditions (14.98%) were significant. This proportion is lower than that of *SI* of firing rate (30.40%, Fig9). Like firing rate, the proportion of significant *SI* of neural latency was relatively higher at short S1–S2 distance and decreased as the S1–S2 distance increased (19.56% for 2∼4 mm, 13.75% for 4∼6 mm, 12.5% for 6∼8 mm, and 8.33% for 8∼10 mm of S1–S2 distance in cortical dimension).

## Discussion

### Results Summary

In the current study, we assessed the possibility that V1 spike activity is involved in encoding spatiotemporal sequences of oriented stimuli encompassing spatial locations in and out of the classical receptive field. We reasoned that a critical requirement for encoding spatiotemporal sequences is the encoding of temporal intervals between sequential stimuli. The time course of surround suppression by onset or offset of surround stimulus has been previously examined [9]. In their experiments, the RF stimulus appeared first and the latency and time course of suppression of the on-going RF response by a subsequently delivered annular stimulus was measured. In contrast, in the current study, we focused on the effects of sequentially delivering stimuli that were non-overlapping in time at various SOA. An intriguing finding of the current study is that the neural activity of V1 is modulated by the temporal interval of focal stimuli based on surround interaction; the responses of single neurons to sequential presentations of focal oriented stimuli in and outside the RF were modulated in a way that depended on the temporal intervals between the two stimuli. The dependency of modulation on SOA can be summarized in three aspects. First, strong modulation occurred at a specific SOA which was variable across S1 positions or cells, and thus, modulations at short SOAs were not always larger than those at larger SOAs (Fig. 3). Second, for some cells, modulation tended to be larger for shorter SOAs compared to larger SOAs (Fig. 5). Thus, for these cells, response modulation had a component that can be described as a monotonic decay with SOA. Third, the modulation had a periodic component across SOA (Fig. 6). Thus, there appears to be multiple mechanisms by which the temporal interval of sequential events can be related to V1 activity. These results suggest that V1 neurons are sensitive to temporal linkage of spatial events inside and outside the RF, thereby the response selectivity of V1 neurons can be extended to spatiotemporal dimension of stimuli encompassing inside and outside the RF. This is consistent with the increase in response selectivity and sparseness by surround stimuli [5], [6]. The results obtained in the current study, although focal stimuli were presented in RF surrounds at varying SOA, are also consistent with and extend previously-reported surround interactions [7], [8], [9], [10], [11], [35].

In our further study [14], we trained monkeys to discriminate the temporal interval between two stationary Gabor stimuli, the first one outside the RF (S1) and the second one in the RF (S2), as in the current study. The SOA between S1 and S2 was randomly chosen from three intervals, ‘short’, ‘intermediate’, and ‘long’ in the range of tens of milliseconds. Reward was contingent upon saccadic eye movement made to one of two targets that appeared later; saccades to the upper one were rewarded in those trials in which the SOA was ‘short’, and saccades to the lower one were rewarded for ‘long’ SOA. In the trials with an intermediate SOA, reward was delivered in randomly-chose half of trials, regardless of the animal’s choice. They observed that V1 spike activity showed an early modulation depending on the sequence interval, and a later modulation depending on behavioral choice. These results suggested that V1 neurons are involved in encoding and discriminating the spatiotemporal sequences of oriented stimuli, based on surround interactions.

We emphasize that SOA-dependency is a property encompassing both focal spatial regions inside and outside the RF, and is thus separate from motion tuning or directional selectivity confined within RF. Muller and colleagues (2003) examined the effects of SOA between RF and surround stimuli, but with a surround stimulus that extended around the RF, completely enclosing it, and found that suppression was the strongest with simultaneous presentation of RF and surround stimuli and gradually decayed as SOA increased up to approximately 100 ms. This is consistent with Fig. 8D, but the SOA-dependency of individual cells deviated from this monotonic dependency on SOA. The visual area covered by S1 in the present study was considerably smaller than the area covered by the enclosing stimulus used by Muller and colleagues; the area of the smallest annulus enclosing S1 was 8 times that of S1 (Fig. 2A). This may be a reason why the magnitude of suppression in the current study is rather weak compared to previous studies reporting as much as an 80% reduction of the RF response for a tightly surrounding annulus [9]. If S1s at different focal locations have different SOA-dependency, an annulus consisting of multiple S1s would produce a complex surround interaction. The monotonic and periodic components described in Fig. 6 is also consistent with this interpretation. When a large extent of surround region is stimulated, the periodic SOA-dependency may disappear because of its dependency on S1 position and thus annihilation among different surround regions. In contrast, the SOA-dependency by the monotonic component which is relatively independent of S1 position persists. The rapid decay of surround modulation with SOA under large surround targets is consistent with the dominance of monotonic component at short SOAs.

The number of unique sequences of Gabor stimuli is immense, given the combinatorial explosion of possible configurations contributed by their size, orientation, distance, SOA, spatial frequency, and phase relationship. It is thought that modulation of spike response to RF stimulus depends on combinations of orientation and spatial location of surround stimulus with respect to the RF stimulus [10]. Similarly, a wide-field natural scene [6], [38] is likely to include many of these sequences, and it is not difficult to imagine that surround interaction involves a complex interaction among these sequences. We tested only a subset of these sequences because of the limited number of trials that can be tested within a single recording session. Nevertheless, it is clear that spike response to S2 was modulated by SOA, suggesting that V1 neurons are selective for the sequence of oriented stimuli that are separated in space and time.

### Relation to Temporal Interval Encoding

While the central mechanisms for spatial aspects of visual stimuli have been relatively well understood, few experimental studies have examined how and where in the visual pathway the information regarding temporal interval is encoded. Buonomano studied the timing and propagation of neural responses in cortical slices based on local cortical networks, and reported that in response to a single electrical stimulation, the network exhibited a reproducible temporal pattern of activity in a fixed latency up to few hundred milliseconds [39]. Series and colleagues hypothesized that feedforward and long-range horizontal connections in the primary visual cortex (V1) may underlie the computation of spatiotemporal sequences that are necessary for speed perception [40]. Their model predicted that V1 responses to an oriented stimulus presented in the RF could be modulated by another oriented stimulus presented a few tens of milliseconds earlier outside the RF. Thus, it is likely that the stimulus outside the RF primes a dynamic change in the state of the cortical network [41], [42] by the time the RF stimulus arrives, and that the changed cortical state modulates the response to the RF stimulus in a manner that depends on the temporal interval between the two stimuli. It has been suggested that low level sensory neurons that are tuned for various temporal delays [43], [44] may recover temporal intervals between spatial events, or that interval selectivity can be derived by simple networks without explicit timing elements [45]. The results obtained in the current study are consistent with the idea of processing temporal interval dealt with in these studies; SOA-dependency of V1 spike activity based on surround interaction may contribute to processing temporal interval.

### Relation to Motion Processing

**Figure 10:**
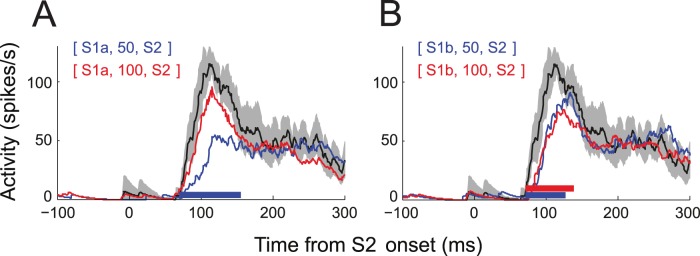
Effects of stimulus speed. This cell is the same as Fig. 5. Time courses of spike response of the cell for S1 at one RF diameter away from S2 (A) and two RF diameters away (B) conditions. Shown in each panel are mean spike density traces for S1–S2 sequence with SOA of 50 ms (blue) and 100 ms (red). Mean spike density for the S2-alone condition is also shown for comparison (black) with its 95% confidence interval (mean±2 SEM, gray shade). All these stimulus conditions, including other SOA conditions, were randomized within the same block during data collection. Note that the peak spike response to S1a–S2 sequence with SOA of 50 ms (blue trace in A) was reduced by half compared to response to S2 alone (black). Also note that the magnitude of this response is quite different from that for S1b–S2 sequence with SOA of 100 ms (red trace in B), although the apparent motion speed of these two conditions is roughly the same.

Spatiotemporally close stimulus sequences cause apparent motion, in which the temporal interval between spatially-displaced sequential targets and perceived motion speed are closely related to each other. The response to sequential stimuli inside and outside the RF has been implicated for motion processing, and a corresponding model has been proposed [40]. Thus, varying SOA means changing motion speed as well. However, it appears that SOA-dependency is not simply related to speed tuning for the following reasons. If the magnitude of the neural response to a stimulus sequence is related to motion speed as in other areas [46], the SOA associated with the peak neural response may vary proportionally to the distance between S1 and the RF, because for a given speed, a doubling of the spatial interval must be accompanied by a doubling of the temporal interval. However, it appears that this was not the case (Fig. 10). Also, the SOA-dependency, which often shows modulation of neural response at multiple SOAs appears to be not compatible with the idea of facilitation or suppression linked to linearly-scaled underestimation or overestimation of motion speed.

### Potential Mechanisms for SOA-selectivity

The mechanism by which SOA affects the modulation of activity by S1 is a matter of speculation. One possibility is an interaction between the input from the RF stimulus and a cortical state that undergoes modification by a preceding event [41]. Thus, the thalamic input mediating S2 stimulation interacts with a feedforward, lateral or feedback input mediating S1 stimulation that does not evoke spike responses by itself. The state dependency of cortical response has been experimentally investigated. Spike responses to preferred stimulus are modulated by preceding stimuli with a short temporal interval in visual [13], [47], [48] and auditory [49], [50], [51] cortices. In relation to this, the neural response of visual cortex can encode previously presented stimuli [52], and monkey auditory cortex neurons can be highly selective to the sequence of tones [53]. This state-dependency of neural response can be a mechanism to increase selectivity of cortical neurons; for example, spatial selectivity of V1 neurons for stimulus orientation can be refined to encode spatiotemporal sequences of oriented stimuli that are separated by a spatial distance and a temporal interval. Thus, we propose that a role of surround interaction in V1, in addition to its known roles such as contour integration [2] or perceptual filling-in [54], is related to encoding spatiotemporal sequence of oriented stimuli inside and outside the RF.

What would be the anatomical substrates that mediate such a state change? Long-range horizontal connections are one clear candidate. A brief focal visual stimulation elicits a spreading wave of activity in cortex [32], presumably through long-range horizontal connections radiating from the stimulation site [55], and causes fluctuation of local field potentials [56] and intracellular potentials [33] in nearby neurons after a temporal delay that depends on the distance between the stimulation site and the neuron’s RF [33]. The periodic SOA-dependency of some cells (Fig. 6D), its apparent phase-dependency on S1 position and the estimated propagation speed (Fig. 6F) suggest that the periodic component is mediated by horizontal connections. However, the modulation of response at a multiple SOAs may result from different paths mediating surround interactions. Modulation of activity at an SOA of 0 ms, even from distant S1, indicates that circuits other than intrinsic horizontal connections, such as geniculo-cortical feedforward [12], [57], [58], [59], [60] and/or extrastriate feedback connections [61], [62] may also mediate the S1 effects, each at different SOA. For the neurons in our sample, the SOAs associated with the most frequent suppression were around 10–20 ms and 40–60 ms (Fig. 8B), making the magnitude of suppression variable depending on SOA within the modulation window. This discontinuous pattern across SOA in suppressive modulation may reflect multiple pathways mediating surround interaction. Alternatively, it may reflect our use of discrete units of distance between S1 and S2 in units of RF diameter without assessing intermediate distances. The distribution of eccentricities of RF centers was bimodal because those obtained from monkey DC were smaller than those from monkey CR. However, the pattern of SOA-dependent significant modulation was similar in both animals, and in particular, the more frequent suppression around SOA of 10–20 ms and 40–60 ms that result in a discontinuity in the modulation pattern was present in both animals. Therefore, we conclude that the discontinuous pattern of SOA-dependency was not related to the distribution of RF eccentricity of sampled neurons.

Thus, the relationship between preferred SOA and spatial distance between S1 and S2 is not straightforward. In any case, SOA-dependency found in the current study suggests a novel role of surround interaction, and is consistent with spatially-localized temporal processing [63] and reliable timing based on networks [39].

### Readout of Temporal Interval

Finally we consider computational challenges related to readout mechanisms of temporal interval. We speculate that, as an obvious candidate, downstream coincident detectors receiving oriented inputs from both neuron pools representing S1 and S2 will be able to decode the temporal interval between S1 and S2 based on the temporal interval in latency or peak between the activities of the two neuron pools. This may provide more reliable information than the modulation of response to S2. What we consider here are the issues related to the possibility that V1 neurons themselves encode temporal interval based on surround interaction. First, the SOA that was associated with a strong modulation of neural response was often not singular. The response modulation at multiple SOAs cannot be used as a simple measure of temporal interval. One clear candidate for the extraction of such information is based on pooling the activity across an active neural population. An example is the owl’s space-specific inferior colliculus neurons, which are modulated by regularly repeated interaural time differences [64], [65].

Second, regarding neural mechanisms of SOA-dependency, single-neuron responses to S1–S2 sequence are not described by a simple linear (weighted) sum of SOA-adjusted consecutive responses to S1 alone and S2 alone, because single cells do not discharge spikes to S1. This is contrasted with population response in which a sequence of full-field oriented stimuli can be approximated by a linear combination of consecutive responses to the individual stimuli in the sequence for readout of stimulus orientation [48].

Third, from a computational point of view, readout mechanisms of temporal information by downstream neurons will be simpler with facilitative than with suppressive modulation by S1. In our experimental condition, suppression was dominant for the initial transient response and for collinear S1 configurations, whereas facilitation typically occurred later and was dominant for parallel S1 configurations. This may not be a serious problem if the increase in the selectivity of spike response by surround suppression [5], [6] is related to encoding of temporal interval. This is also true for facilitative modulation by S1, which was less common than suppression. As can be seen in Fig. 3B, S1 alone occasionally suppressed activity about 100 ms after its onset, and activity increased after this suppression (best seen in S1c alone condition). Thus, facilitation at around 150–200 ms (Fig. 3F) is likely to be a rebound after suppression. However, if facilitation simply reflects a rebound from suppression, the relative dominance of facilitation for parallel S1 configuration (Fig. 8) is not to be expected. Additional mechanisms of response modulation would be the arrival of suppressive and facilitative signals with different delays. For some cells, facilitative inputs occurred for longer SOAs than did suppressive ones (not shown). Therefore, response modulation, whether facilitative or suppressive, may account for response selectivity.
